# Different deployments of attentional breadth selectively predict UFOV task performance in older adults

**DOI:** 10.1186/s41235-024-00569-3

**Published:** 2024-06-26

**Authors:** Nicholas J. Wyche, Mark Edwards, Stephanie C. Goodhew

**Affiliations:** School of Medicine and Psychology (Building 39), Australian National University, Canberra, ACT 2601 Australia

**Keywords:** Useful field of view, Attention, Attentional breadth, Individual differences

## Abstract

The Useful Field of View task (UFOV) is a strong and reliable predictor of crash risk in older drivers. However, while the functional domain of attention is clearly implicated in UFOV performance, the potential role of one specific attentional process remains unclear: attentional breadth (the spatial extent of the attended region around the point of visual fixation). The goal of the present study was to systematically test the role of two distinct aspects of attentional breadth, maintaining a specific breadth of attention and resizing the attended region, in UFOV performance. To this end, 135 older adults completed the UFOV and modified Navon tasks to measure their efficiency in maintaining, contracting, and expanding the breadth of attention. We then examined individual-difference associations between these aspects of attentional breadth deployment and UFOV performance. We found that performance on UFOV Subtask 2 was associated with efficient contraction of attentional breadth (i.e., resizing the attended region to a smaller area), while Subtask 3 performance was associated with the efficiency of expanding attentional breadth (i.e., resizing the attended region to a larger area). The selectivity of these relationships appears to implicate these specific deployments of attentional breadth in how people complete the task, as it suggests that these relationships are not simply attributable to shared variance in a broader domain of cognitive functioning. The implications of these results for our understanding of UFOV, as well as future research directions that test the relative contributions of different cognitive processes in predicting task performance, are discussed.

**Supplementary Information:**

The online version contains supplementary material available at 10.1186/s41235-024-00569-3.

## Introduction

As many countries have begun to engage with the policy implications of ageing populations, driver safety in adults over age 60 (*older adults*) has become an important research concern. A focus of this research has been the development of tools which permit both assessment and training of competency in older drivers. One such tool that has gained widespread use is the Useful Field of View task (UFOV; Ball & Owsley, 1993). While UFOV has clear applied value as an assessment and training tool (Ball et al., 2002b; Clay et al., 2005; Cross et al., 2009; Edwards et al., 2005a, 2005b; Horswill et al., 2010; Rubin et al., 2007; Vance et al., 2007), it is also important to identify the cognitive processes involved in task performance. There is broad agreement that the functional domain of attention is involved in UFOV performance (Anstey et al., 2012; Hoffman et al., 2005; Matas et al., 2014; Woutersen et al., 2017), but the potential role of one specific process remains unclear: attentional breadth (the spatial extent of the attended region around the point of visual fixation; Goodhew, 2020). UFOV was initially understood to measure the ‘constriction of spatial attention’ (Ball & Sekuler, 1986) and is occasionally used explicitly as a measure of attentional breadth (Gray et al., 2014), but the validity of this usage has never been empirically confirmed. Indeed, the limited research investigating the relationship between attentional breadth and UFOV has not found support for this association (Cosman et al., 2012a). The goal of the present study was to systematically test two distinct aspects of attentional breadth, namely the ability to maintain a specific breadth of attention and the efficiency of resizing the extent of the attended region, as potential correlates of UFOV performance. In the following sections, the rationale for understanding UFOV from a theoretical perspective is outlined, and the task and the cognitive processes it is traditionally understood to represent are then considered through a critical lens. The available research into the relationship between attentional breadth and UFOV performance is then reviewed, leading to an explanation of why attentional breadth may be implicated in UFOV task performance.

### Why understand UFOV from a theoretical perspective?

UFOV has become an enduring feature of the driver safety literature because of its largely unsurpassed ability to not only predict crash risk in older adults, but also to function as a training tool which can help older adults to improve their driving capabilities. UFOV has been shown to predict self-reported incidence of crashes in older adults (Ball et al., 1993; Goode et al., 1998; Horswill et al., 2010; Owsley et al., 1991), as well as prospective crash risk (i.e., incidence of real-world crashes in a time period following administration of UFOV; Cross et al., 2009; Owsley et al., 1998; Rubin et al., 2007). UFOV also predicts ability to perceive hazards in a video-based driving task (Horswill et al., 2008), as well as performance in both simulated driving (Allahyari et al., 2008; Bélanger et al., 2010; Rizzo et al., 1997) and real-world driving scenarios (Classen et al., 2013; Huisingh et al., 2017; Myers et al., 2000; Selander et al., 2011). Indeed, three separate meta-analyses have concluded that UFOV robustly and consistently predicts driving performance in a range of settings (Clay et al., 2005; Mathias & Lucas, 2009; Stefanidis et al., 2023). Another advantage of UFOV is that it also predicts when older drivers will have difficult with specific real-world driving settings, such as entering into traffic (Pietras et al., 2006), turning across an intersection (Rusch et al., 2016), dealing with distractions while driving (Wood et al., 2012), and dealing with unexpected stimuli (Krasniuk et al., 2022). Finally, a unique aspect of UFOV’s utility is its potential as an intervention—training on variants of the task improves driving performance in older adults (Ball et al., 2002a; Edwards et al., 2005a, 2005b; Vance et al., 2007), and this improvement is reportedly maintained for as long as ten years after initial training (Edwards et al., 2018; Rebok et al., 2014).

Given that UFOV so successfully serves the dual purposes of predicting and improving real-world driver safety outcomes in older adults, why does it matter whether we understand exactly what cognitive and perceptual processes the task measures? We believe that understanding the theoretical processes involved in UFOV performance is important for two reasons. Firstly, elucidating the mechanisms of UFOV performance allows us to better understand the perceptual and cognitive factors which are linked to crash risk for older adults. Secondly and consequently, knowledge about cognitive factors involved in driver safety may lead to the development of even more effective prediction and intervention tools which test and train specific cognitive competencies, as well as the development of technological solutions which are designed to alleviate specific deficits in cognitive processing during driving (e.g., Carr & Grover, 2020). Therefore, although UFOV’s applied utility represents the core of its value proposition, it is also worthwhile to investigate the attentional processes involved in UFOV performance. The following sections consider the design of the UFOV task and the current state of knowledge about the spatial–attentional processes implicated in UFOV performance, before treating the conflicting evidence about the role of attentional breadth in task performance.

### Spatial–attentional processes involved in UFOV

Most implementations of UFOV consist of three subtasks presented in increasing order of difficulty (Fig. 2). UFOV uses a temporal threshold, specifically the stimulus duration at which the participant can achieve approximately 75% performance on the task as assessed using a staircase procedure. All subtasks involve the central presentation of a full-contrast target stimulus (a simplified line drawing of either a car or a truck) presented in the centre of the screen surrounded by a box, while Subtasks 2 and 3 also present a peripheral target stimulus (a line drawing of a car of equal size and contrast to the central stimulus) which is not surrounded by a box. In Subtask 1 (the 'processing speed' paradigm), the central stimulus must be identified as either a car or a truck. In Subtask 2 (the 'divided attention' paradigm), the central stimulus is shown, as well as the peripheral target stimulus which is simultaneously displayed in one of eight locations at equidistant eccentricities from the central image. Participants are asked to identify the central stimulus as for Subtask 1 and then to identify the location of the second stimulus. Subtask 3 (the 'selective attention' paradigm) is identical to Subtask 2, with the peripheral target appearing in one of the same eight locations. However, the target is now surrounded by distractor stimuli which must be ignored, positioned in three concentric rings with the target appearing in the outermost ring.

The design and conceptualization of the UFOV subtasks have both evolved considerably since the task’s inception. UFOV was originally designed as a measure of ‘constriction of spatial attention’ in older adults (Ball & Sekuler, 1986), with peripheral stimuli in Subtasks 2 and 3 presented at a range of eccentricities. These stimulus displays were understood to gauge the ‘useful’ or ‘functional field of view’, the area over which information can be obtained in a single fixation without head or eye movements (i.e., without any change in the location or size of the focus of attention; Sanders, 1970). Assuming this understanding to be correct, these tasks might be understood to gauge a *maximal* attentional breadth (i.e., the broadest area which the attended region can be extended to cover), and therefore poor task performance reflects a constriction of attentional breadth. Subsequent findings have challenged the idea that UFOV gauges a constriction of spatial attention (Seiple et al., 1996; Sekuler et al., 2000), instead conceptualizing the task as a metric of cognitive 'processing speed' in older adults (Wood & Owsley, 2014). In accordance with this view, stimuli for Subtasks 2 and 3 are now presented only at a single eccentricity, and in this format the task has been standardised and marketed commercially for driver assessment purposes (UFOV®; Visual Awareness, Inc., Punta Gorda, FL).

While attention as a broad domain of functioning has frequently been implicated in UFOV performance, identification of the specific *processes* of spatial–attentional deployment that are involved has remained challenging. The following section considers the available evidence on the relationship between attentional breadth and UFOV performance, concluding that while the ability to set a minimal breadth of attention may not be related to UFOV, there is reason to think that setting a maximal breadth of attention and/or *resizing* the breadth of attention could be implicated in task completion.

#### UFOV and setting a fixed breadth of attention

Although earlier versions of UFOV which varied stimulus eccentricities may have gauged the ability to adopt a maximal breadth of attention (i.e., the largest breath of attention usable by an individual), the current versions of Subtasks 2 and 3 use stimulus arrays presented at only a single eccentricity. Therefore, UFOV might gauge the ability to set and maintain a breadth of attention that encompasses these stimulus displays. This possibility was investigated by Cosman et al. (2012a), who concluded that impaired UFOV performance was not linked to a constricted breadth of attention as gauged by a Flanker task.

However, this use of the Flanker task presents some difficulties of interpretation regarding the role of attentional breadth in completion of UFOV. The classical Flanker paradigm (Eriksen & Eriksen, 1974) presents a series of trials where a letter presented at central fixation is ‘flanked’ by distractor letters, and participants are asked to identify which of two target letters is presented in the central position. The key manipulation is target-flanker congruency: the distractors can either be identical to the target (the congruent condition) or the other possible target letter (the incongruent condition). If a large Flanker effect (i.e., the RT difference between congruent and incongruent trials) is observed, it is assumed that the participant has adopted a large breadth of attention such that the distractors are falling within the attended region and interfering with identification of the central letter on incongruent trials. Conversely, Cosman et al. (2012a) predicted that if UFOV performance is linked to a constriction of attentional breadth, participants who performed more poorly on UFOV would show reduced Flanker effects, as the distractors would not fall within their more limited breadths of attention and thus would not interfere with identification of the central letter. Ultimately, results in Cosman et al.’s (2012a) study indicated no difference in the magnitude of the Flanker effect when comparing UFOV impaired and non-impaired participants, and they therefore rejected a role of attentional constriction as a determinant of UFOV performance.

However, while there is thought to be an attentional breadth component to Flanker task performance (Caparos & Linnell, 2010; Lee & Pitt, 2021; Miller, 1991), Flanker interference effects can be minimised when a participant adopts and maintains a small attentional breadth focussed on the target, such that the distractors are effectively excluded from the attended region (Hübner & Töbel, 2012). Therefore, a smaller Flanker effect is not necessarily indicative of pathological constriction of attentional breadth, but instead may reflect a strategic narrowing of attentional breadth to complete the Flanker task. Because UFOV requires participants to localize items presented peripherally as well as identifying a central target, a broader, rather than a narrower attentional breadth may be optimal for this task. If so, then this discrepancy between optimal deployments of attentional breadth for the Flanker versus UFOV tasks may explain why the magnitude of Flanker effects was not associated with UFOV performance (Cosman et al., 2012a). Overall, a relationship between attentional breadth and UFOV performance cannot be ruled out based on these findings alone, and there is still value in assessing whether setting a *larger* breadth of attention is linked to task performance.

#### UFOV and resizing attention

**Fig. 1:**
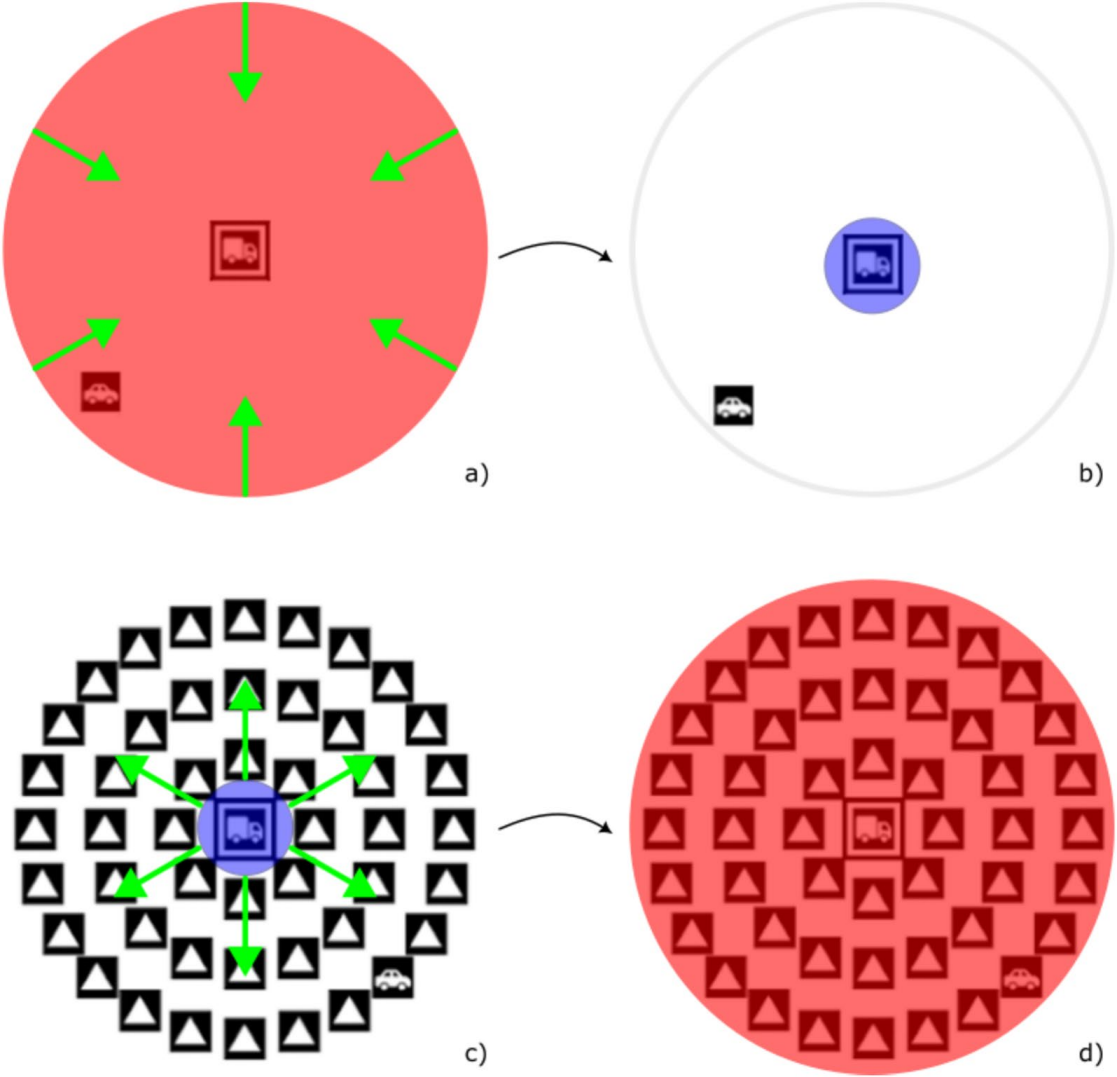
Possible resizing of attentional breadth when completing UFOV subtasks. UFOV Subtask 2 is depicted in (**a**)–(**b**), and UFOV Subtask 3 is depicted in (**c**)–(**d**). For UFOV Subtask 2, participants may (a) initially adopt a large breadth of attention (red circle) that encompasses the whole display to locate the peripheral target, before contracting the breadth of attention (green arrows) to (**b**) focus on a small region of the display (blue circle) and identify the central target. Conversely, for Subtask 3, participants may (**c**) initially adopt a small breadth of attention (blue circle) to identify the central target, before expanding the breadth of attention (green arrows) and (**d**) perceiving the whole display in an attempt to locate the peripheral target. These are only two possible mechanisms for task completion; others are described in-text

Even after considering that adopting a large breadth of attention may be linked to UFOV performance, a broader question remains: is this the most likely way that individuals deploy spatial attention when completing the task? One of the most important features of attentional breadth is the trade-off between perceptual acuity and size of attended region, often labelled the zoom-lens model (Eriksen & St. James, 1986). When attentional breadth is smaller, perceptual acuity is strong and fine details are easier to perceive, but when attentional breadth broadens, there is a corresponding decline in perceptual acuity (Sasaki et al., 2001). The zoom-lens model implies the necessity of *resizing* attentional breadth as it is deployed for different information acquisition purposes (see Fig. 1). While earlier research into attentional breadth focused on adoption and maintenance of specific breadths, more recent research has demonstrated dynamic rescaling of the size of the attended region (Calcott & Berkman, 2014; Goodhew, 2021; Goodhew & Plummer, 2019). Further, individuals are known to vary significantly in the time taken to resize the breadth of attention: some demonstrate almost no time cost during resizing, while for others it is a slower process (Goodhew, 2021).

How might resizing of attentional breadth be implicated in UFOV Subtasks 2 and 3? Both subtasks require identification of a small central stimulus (car or truck), a task which requires fine spatial acuity and thus a small breadth of attention. However, both subtasks also require acquisition of information about the location of a peripherally presented target. Subtask 2 requires only localisation of the peripheral target: as it is presented without distractor items, no information about target identity is needed, and fine perceptual acuity is not necessary for task completion (Tsal & Bareket, 1999). Therefore, during Subtask 2 completion, attentional breadth might initially be focussed narrowly on the centre to identify the central target and then expanded to encompass the entire display (Fig. 1), or contracted to the central item after initial localisation of the peripheral stimulus, without the need for a shift of attention (Goodhew & Plummer, 2019). In Subtask 3 however, the peripheral target is embedded in a distractor array and may be less distinguishable from these distractors under circumstances of low perceptual acuity. While this would normally lead to a visual search using shifts of attention to locate the target item, this is less feasible in UFOV due to the limited display durations for each stimulus array (ranging between 16 and 500 ms), which inhibit planning and execution of serial eye movements. These time constraints may force individuals to rely on expansion of attentional breadth after initial identification of the central target (Fig. 1) to minimise the role of crowding effects (Matas et al., 2014), or an unchanging adoption of a large attentional breadth, to acquire information about the potential location of the peripheral target even when many distractors are present.

### Present Study

Overall, while it is unlikely that adoption of a minimal breadth of attention is implicated in UFOV performance, two mechanisms which may more plausibly be involved in task completion are setting a large breadth of attention, and/or the *resizing* of attentional breadth. The goal of the present study was to test both static (setting a specific breadth) and dynamic (resizing breadth) attentional breadth accounts of UFOV performance.

## Methods

### Participants

The planned analysis was a linear multiple regression assessing the relationship between three predictors [(1) Navon preference score: comparison of ability to set small and large breadths of attention, (2)–(3) attentional breadth resizing: Navon expansion score, Navon contraction score] and the criterion of UFOV performance in a sample of older adults. An a priori power analysis performed in G*Power (Faul et al., 2007) indicated that a sample of 119 was required to detect a medium effect size of *f*^2^ = 0.15 (Cohen, 2013) with power of 95% in a three-predictor linear regression. Anticipating participant exclusions, an extra fifteen participants were sought for the study, bringing the planned sample size to 134; 135 participants ultimately completed the study due to the way that the recruitment platform implemented study enrolment cut-offs. All participants were located in the UK and recruited via the Prolific platform, with the eligibility criteria that they were age 60 or older and used a computer (rather than a tablet or phone) to complete the study.

Demographic information for the sample is reported in Table 1. The most common country of birth was the UK (*n* = 118). The Cognitive Failures Questionnaire (CFQ; Broadbent et al., 1982), a measure of the frequency of errors that participants made in activities of daily living, was also administered. CFQ results have previously been linked to car accident risk (Wallace & Vodanovich, 2003), so it was administered to provide converging evidence that the sample did not have anomalously high or low accident risk, factors which would affect our interpretation of the sample’s UFOV scores. Descriptive statistics for the CFQ in this sample (*M* = 32.2, SD = 11.0) were similar to those previously reported for a normative sample of 65–74-year-olds (*M* = 31.2, SD = 11.2; Knight et al., 2004). By contrast, this sample’s descriptives for UFOV composite score (*M* = 190.6, SD = *74.3),* a commonly computed metric which sums each individual’s performances for UFOV Subtasks 1–3, were significantly better than those reported in normative data (*M* = 481.93, SD = 247.53; Edwards et al., 2005a).

**Table 1:** Demographic Characteristics and Driving Experience of Sample

Characteristic	*n*	*M*	SD	Range
Gender				
Female	63			
Male	67			
Handedness^a^				
Left	11			
Right	115			
Ambidextrous	2			
English as first language?				
Yes	128			
No	2			
Highest educational attainment				
Some high school	6			
Completed high school	45			
Professional or trade qualification	6			
Diploma	4			
Undergraduate degree	45			
Postgraduate degree	24			
Driving experience				
Never held driver’s licence	14			
No current driver’s licence	7			
Full licence	109			
Age		65.5	4.6	60–81
Days driven per week^b^		3.6	2.1	0–7
Years held driver’s licence^b^		43.3	7.7	20–65
CFQ score^c^		32.2	11.0	6–69
UFOV composite score^d^		190.6	74.3	64.8–461.1

For older adults, total *N* = 135; one extra participant was recruited due to the way that Prolific implements study enrolment cut-offs. Five participants did not provide any demographic data, so *n* for above reports is 130 unless noted below

^a^Two participants did not enter a response about their handedness

^b^For Days Driven Per Week and Years Held Driver’s Licence, one participant did not enter responses despite holding a current full licence

^c^For 14 participants, responses to CFQ survey were incomplete, so their partial responses were not included in calculation of these descriptive statistics

^d^UFOV composite score (summed UFOV Subtask 1–3 scores, in ms) is a common metric of UFOV performance which is a strong predictor of real-world driving outcomes

### Procedure and experimental stimuli

All ethical aspects of the experiment were approved by the Australian National University’s Delegated Science and Medical Human Research Ethics Committee (protocol 2022/029). All participants provided informed consent before the experiment and were able to withdraw at any time without penalty by closing the program window. Participants were compensated with a payment of £13.50.

Participants completed the experiment remotely on their own computers using the Inquisit 6.6.1 program in May of 2023. As differences in screen size and viewing distance made it impossible to ensure that stimuli subtended the same visual angle for all participants, stimulus sizes were scaled to occupy the same percentage of screen dimensions across devices. Participants familiarized themselves with each task using a self-paced introduction and practice block (a small number of trials with feedback provided). For all tasks using Navon figures, if a prescribed practice threshold of 75% accuracy was not met, participants repeated the practice task. When the prescribed performance threshold was met, participants proceeded to the experimental block(s) for the task. After completing all tasks, participants completed a demographic survey and then the CFQ.

All participants completed five tasks in total: (1) UFOV, (2) Navon all-global block (measuring ability to set a large breadth of attention), (3) Navon all-local block (measuring ability to set a small breadth of attention), (4) Navon expansion (measuring ability to expand attentional breadth), and (5) Navon contraction (measuring ability to contract attentional breadth). Participants were randomly assigned to one of eight running orders for these tasks, which are described in the Supplementary Materials.

#### UFOV task

**Fig. 2:**
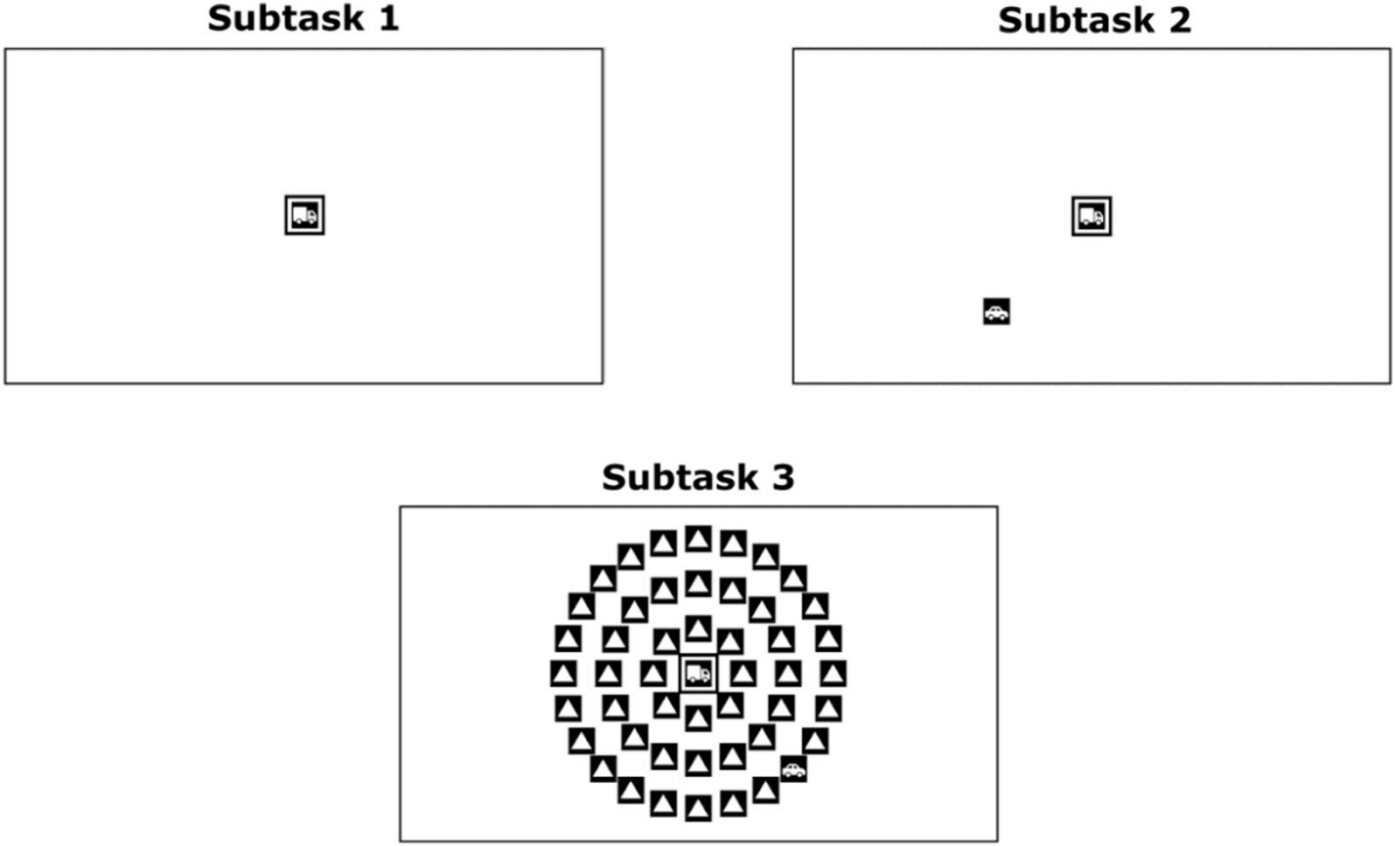
Sample stimulus displays for the three subtasks of UFOV. These displays illustrate proportional stimulus sizing on a 1980 × 1020 monitor (screen dimensions indicated by black box)

A conceptual overview of UFOV’s design is presented in the Introduction. Regarding specifics of stimulus presentation, for Subtask 1 an image of a car or truck subtending 1.8 by 1.8° of visual angle^1^ was displayed in the centre of the screen, surrounded by a black box subtending 2.0 by 2.0°. The initial display time was 200 ms. Participants were instructed to identify this image as accurately as possible by clicking either a car or a truck icon that appeared on the screen after the trial display finished. For Subtask 2, an additional image of a car of the same size as the central stimulus was displayed at one of eight possible locations along the circumference of a circle with a radius of 13.7° of visual angle. To input the location of the outer car, a screen then appeared with a clickable button in each of the eight possible response locations, and the participant was instructed to click the mouse in the location where the outer stimulus appeared. Subtask 3 operated identically to Subtask 2, except for the addition of three concentric rings of triangles located along the circumferences of circles with radii of 4.6°, 9.1° and 13.7° of visual angle. The outermost ring of triangles lay along the same circumference as the outer stimulus. Sample UFOV stimulus displays used in this study can be seen in Fig. 2. For all subtasks, a random dot mask was shown for 1000 ms after the display of the trial stimulus.

For all subtasks, a staircase design was employed to determine the threshold display time at which participants could perform at 75% accuracy. A staircase is an adaptive procedure in which the display times used change according to participants’ performance. Here, the staircase was a 2-down 1-up design with a step interval of 16.67 ms and a starting point of 300 ms: participants had to respond correctly on two consecutive trials for the subsequent display time to decrease by one step, but only one incorrect response was required to increase the subsequent display time by one step. To quickly approximate a threshold level of performance, a step size of 66.67 ms was employed until the first reversal. Each subtask terminated after one of three conditions was satisfied: (1) three consecutive correct responses at the lowest display time (16.67 ms), or (2) three consecutive incorrect responses at the highest display time (500 ms), or (3) nine reversals in the staircase occurring.

Each subtask was scored based on which termination condition was met. If the first or second termination condition was met, the participant’s UFOV subtask score was taken as 16.67 ms or 500 ms, respectively. If the third termination condition was met, the participant’s UFOV subtask score was the average of the display times at the reversal points. In the UFOV paradigm, a lower score indicates better performance, as it reflects the ability to complete the required task when a shorter display time is used.

### Measuring attentional breadth

*Navon Stimuli* We chose the Navon task as a measure of attentional breadth because there is good evidence for its validity as an operationalisation of this construct: neuroimaging evidence confirms that when attending to the local elements of Navon figures, the region of activation in the primary visual cortex is narrowed, and when attending to global elements, it is broadened (Sasaki et al., 2001). As described below, the Navon All-Local and All-Global tasks were included as operationalizations of participants’ ability to instantiate specific breadths of attention, while Navon Expansion and Contraction tasks were used to measure participants’ resizing efficiency.

**Fig. 3:**
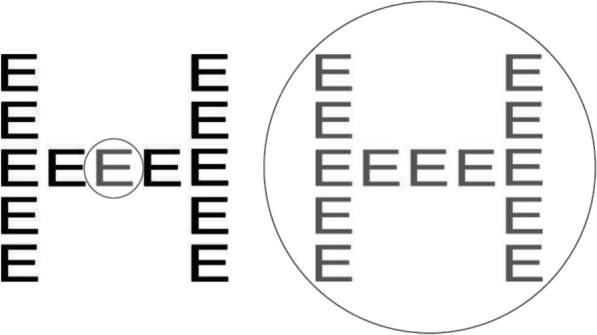
Breadths of attention used in completing the Navon task. At left, the observer adopts a small breadth of attention to efficiently perceive the local (component) level of detail (i.e., the letter E). At right, the observer adopts a large breadth of attention to perceive the global (composite) level of detail (i.e., the letter H). To perceive information at the other level of detail requires resizing of the breadth of attention: for the figure at left, the attended region would be expanded to perceive the global level of detail, while for the figure at right, the attended region would be contracted to perceive the local level of detail. This diagram is for illustrative purposes only: figures are not to scale, and note that E was not a target letter used in this experiment

All the Navon tasks used a stimulus set of eight Navon letters (see Fig. 3 for an example): composite figures depicting a large (global) letter whose shape is made up of a repeated small (local) letter (Navon, 1977). Using the nomenclature ‘GLOBAL–local’, the stimulus set of Navon letters used was: H-e, H-f, T-e, T-f, E-h, E-t, F-h, F-t. One of these Navon letters was shown in the centre of the screen per trial. The global size of each Navon letter was matched to the size of UFOV Subtask 3 arrays, while the size of local Navon letter components was matched to the size of individual UFOV car/truck elements. This size matching ensured that the breadths of attention being instantiated or resized in these tasks corresponded to the physical properties of the UFOV stimuli, in an effort to maximise the possibility of detecting any role of attentional breadth in task completion.

*Navon All-Local and All-Global Tasks* For the All-Local task, participants were instructed to identify whether the letter presented at the local level was ‘T’ or ‘H’, while for the All-Global task, participants were instructed to identify whether the letter presented at the global level was ‘T’ or ‘H’. Participants made their response by pressing the corresponding keys on the keyboard. For each trial, the Navon letter remained on the screen until a response was made, after which the next stimulus was presented following a 1000 ms ISI. For both the All-Local and All-Global tasks, participants completed one block of twenty trials, as reanalysis of published data from Goodhew and Plummer (2019) indicated that this was the minimum number of trials necessary for participants to reliably converge on consistent RTs for these conditions (see Supplementary Materials).

Accuracy performance was measured for the Navon tasks to monitor compliance with task instructions, as anomalously low accuracy was interpreted to represent misunderstanding of the instructions or disengagement from the task. However, the key metric derived from the Navon All-Local and All-Global tasks was a single average RT measure, termed the Navon Preference score, such that:

$${\text{Preference}}\;{\text{Score}} = {\text{RT}}_{{{\text{target}}\;{\text{local}}\;{\text{trials}}}} - {\text{ RT}}_{{{\text{target}}\;{\text{global}}\;{\text{trials}}}}$$

The purpose of calculating the Navon Preference score was to control for between-participant variance on motor RTs and isolate the variance in RT performance which was attributable to instantiation of specific breadths of attention. If raw All-Local and All-Global condition averages were used, variance between participants’ characteristic individual motor RTs (i.e., their general propensity to respond quickly or slowly to a task) and response criteria would be likely to lead to high levels of correlation between these scores. This creates problems of interpretation for two reasons. Firstly, the process of interest (instantiating specific breadths of attention) has not been effectively isolated from other sources of variance. Secondly, a high correlation between All-Global and All-Local scores might lead to difficulties with interpretation of these scores’ respective contributions to the planned regression analyses due to multicollinearity.

Therefore, a Navon Preference Score was calculated for each participant to reflect the relative facility with which they could instantiate the two different breadths of attention tested. A strong positive score indicates that a participant found it relatively more difficult to instantiate a narrow breadth of attention than a broad one, while a strong negative score indicates the inverse. Finally, a score close to zero indicates that a participant had roughly equivalent ability to instantiate broad and narrow breadths of attention.

If Navon Preference Score is *positively* associated with UFOV performance (i.e., *lower* Navon Preference scores are linked to a lower UFOV threshold), this means that participants who are more adept at instantiating a narrow breadth of attention have a performance advantage on UFOV; this would be an unexpected finding as it would contradict the observations of Cosman et al. (2012a) obtained using the Flanker task as described in the Introduction. Conversely, if Navon Preference Score is *negatively* associated with UFOV performance (i.e., *higher* Navon Preference scores are linked to a lower UFOV threshold), this means that participants who are more adept at instantiating a broad breadth of attention have a performance advantage on UFOV; as discussed in the Introduction, this outcome seems more theoretically plausible and therefore more likely.

*Navon Expansion and Contraction Tasks* In the Navon Expansion and Contraction tasks, the target could appear at either the global or local level, and participants were instructed to identify which one of the two target letters (T or H) was present in the stimulus. In the Expansion block, the target was present at the local level on 80% of trials and at the global level on 20% of trials, whereas for the Contraction block, the target was present at the global level on 80% trials and at the local level on 20% trials, where trial types were randomly intermixed.

The two Navon resizing tasks (expansion and contraction) were included as an operationalization of the efficiency of rescaling attentional breadth. When using unequal ratios of global and local trials within the same block, there is evidence that participants adopt an attentional breadth which is optimal for the majority of trials, and must therefore resize the breadth of attention when responding to minority trials (Calcott & Berkman, 2014; Goodhew, 2021; Goodhew & Plummer, 2019). Accordingly, RTs for target identification on minority trials are interpreted to reflect the time to rescale attention away from the breadth set on majority trials. Thus, this modified Navon paradigm operationalizes the rescaling of attentional breadth as an RT score comparing the majority and minority trials in a block.

Following reanalysis of published data (Goodhew & Plummer, 2019) as described above to determine the optimum number of trials, two blocks of 160 trials were administered. Trial timing parameters were identical to the All-Global/All-Local tasks described in the Supplementary Materials. In one block, 80% of trials were target-local and 20% were target-global (majority local block), while in the other block 80% of trials were target-global and 20% were target-local (majority global block). Participants were informed of these ratios before each block commenced. Within these blocks, trial types were randomly intermixed. Ordering of these blocks was determined by the participant’s allocated running order. Rest breaks terminated at the participant’s discretion were offered at multiples of 40 trials (i.e., after 40, 80, and 120 trials).

Two outcome measures were calculated: an *expansion score* from the majority-local block (where attentional breadth must be expanded to respond to minority target-global trials) and a *contraction score* from the majority-global block (where attentional breadth must be contracted to respond to minority target-local trials).

$${\text{Expansion score }} = {\text{ RT}}_{{\text{target global trials}}} - {\text{ RT}}_{{\text{target local trials}}}$$

$${\text{Contraction score }} = {\text{ RT}}_{{\text{target local trials}}} - {\text{ RT}}_{{\text{target global trials}}}$$

These scores are interpreted as measures of the efficiency in resizing attentional breadth: large expansion and contraction costs indicate inefficient resizing. As for the All-Global and All-Local Navon tasks, information about accuracy was collected to check compliance with task instructions.

## Results

### Participant exclusions

Of an original sample size of 135, 28 participants in total were excluded, leaving a final sample size of *n* = 107. These exclusions are described in detail below.

#### Data quality

Some participants’ data were excluded from further analysis because of concerns about data quality. One participant who reported uncorrected visual issues was excluded from further analysis. Seven further participants were excluded for having incomplete or unusable data: six participants had missing data for more than one task, while one participant had a UFOV score of 500 in more than one condition, and due to the remote delivery format of this study, the possibility of a misunderstanding about how to complete the task could not be ruled out.

#### UFOV

No further datasets beyond the single participant described in the ‘Data Quality’ section were excluded on the basis of UFOV performance for two reasons. Firstly, UFOV performance is already bounded by the task’s upper (500 ms) and lower (16.67 ms) display times. Secondly, UFOV uses a staircase design that attempts to identify an individual’s threshold level of performance: given that the dependent variable for each subtask is a single score, trial-level screening analysis is not possible for UFOV.

#### Navon tasks

For the Navon task, the dependent variable was RT. Based on the procedure recommended in Goodhew et al. (2020), RT was first subjected to trial-level analysis for each individual participant: trials were termed *invalid* and removed from further analysis if participants responded so quickly that their responses were likely to be pre-emptive (< 100 ms), or so slowly that they were likely disengaged from the task (> 2.5SDs above participant’s mean RT). For these tasks, invalid responses comprised 5% or less of total responses per participant for all participants, so no datasets were excluded on this basis.

After trial-level screening, participants’ average-level data were screened for outlier cases. Screening for outlier participants was undertaken for accuracy performance using a minimum accuracy threshold of 80% in all Navon task conditions. For accuracy screening, a specified performance threshold was preferred over a z-score criterion due to accuracy being used to check compliance with task instructions (stimuli were shown until response), whereas RT was the primary dependent variable on which variance was expected. On the basis of this accuracy threshold screening, a further 17 participants were excluded from further analysis. This number was unexpectedly high, but may have been attributable to participants’ inability to clarify misunderstandings about task design or controls given the remote delivery format.

Finally, RT screening was applied to all Navon task conditions using a z-score criterion of |z|> 3.29 (Tabachnick & Fidell, 2013). A further two participants were excluded from further analysis.

### Experimental effects for study tasks

Following exclusions, patterns of performance in the remaining sample (*n* = 107) were assessed to establish that group-level experimental effects emerged as expected, an important indicator of construct validity. Task reliability (i.e., the stability of participants’ individual performance levels across time on the study tasks) was also examined for the Navon tasks, with all demonstrating excellent reliability. Additionally, as stimulus sizing was scaled to participants’ screens, a correlational analysis was conducted to assess whether this affected performance on key variables of interest. This analysis did not show any relationship between screen size and task performance (see Table 2 and Supplementary Materials).

**Table 2:** Correlations between UFOV Subtasks and Screen Size

	UFOV subtask 1	UFOV subtask 2	UFOV subtask 3
UFOV subtask 2	.302** [.119, .465]		
UFOV subtask 3	.384*** [.210, .535]	.496*** [.338, .627]	
Screen size	− .182 [− .359, .008]	− .085 [− .271, .106]	− .112 [− .296, .079]

Spearman correlations reported due to non-normality in all variables of interest except UFOV Subtask 3. ** *p* < .01; *** *p* < .001

#### UFOV task

Because UFOV performance is bounded by the display times used in the task, it is important to consider the proportion of the sample performing at ceiling level for each subtask. In other words, if a participant can consistently complete a UFOV subtask at the shortest display time offered by the task, their true UFOV threshold is shorter than the task is able to detect. For Subtask 1, 83% of the sample performed at ceiling level (*M* = 19.1 ms, SD = 9.5 ms), indicating that for most participants their true threshold was outside the bounds measurable by the task. Conversely, for Subtask 2, 11% of the sample performed at ceiling level (*M* = 48.9 ms, SD = 39.5 ms), and for Subtask 3, 0% of the sample performed at ceiling level (*M* = 122.6 ms, SD = 42.2 ms); for these subtasks, either the majority or entirety of participants’ UFOV thresholds fell within the bounds measurable by the task.

As seen in normative data for UFOV (Edwards et al., 2005a), performances on the three subtasks were significantly correlated with each other (see Table 2). Conversely, no significant correlations were detected between screen size and UFOV subtask performance, indicating that the scaling of UFOV stimuli to screen size did not exert systematic influence upon task difficulty.

#### Navon tasks

For the Navon tasks, accuracy performance was high which was expected as the primary individual-difference measures derived from task performance were RT-based (see Table 3). Accuracy performance was compared across task conditions, with results indicating an absence of speed-accuracy trade-offs for Navon task performance (see Supplementary Materials).

**Table 3:** Sample Performance for All Navon Task Conditions

Task condition	Accuracy		RT	
	*M* (%)	*SD* (%)	*M* (ms)	*SD* (ms)
Navon all global	98.7	2.9	595.9	132.9
Navon all local	98.5	2.5	566.3	184.7
Navon preference score	–	–	− 29.6	164.9
Navon contraction				
Global trials	99.5	0.8	647.5	117.5
Local trials	96.0	3.2	897.1	242.6
Contraction score	–	–	249.5	162.7
Navon expansion				
Global trials	97.7	3.1	816.7	160.4
Local trials	99.0	1.0	661.3	130.5
Expansion score	–	–	155.4	112.5

For Navon Contraction, Global Trials was the majority condition and Local Trials was the minority condition. For Navon Expansion, Local Trials was the majority condition and Global Trials was the minority condition

**Fig. 4:**
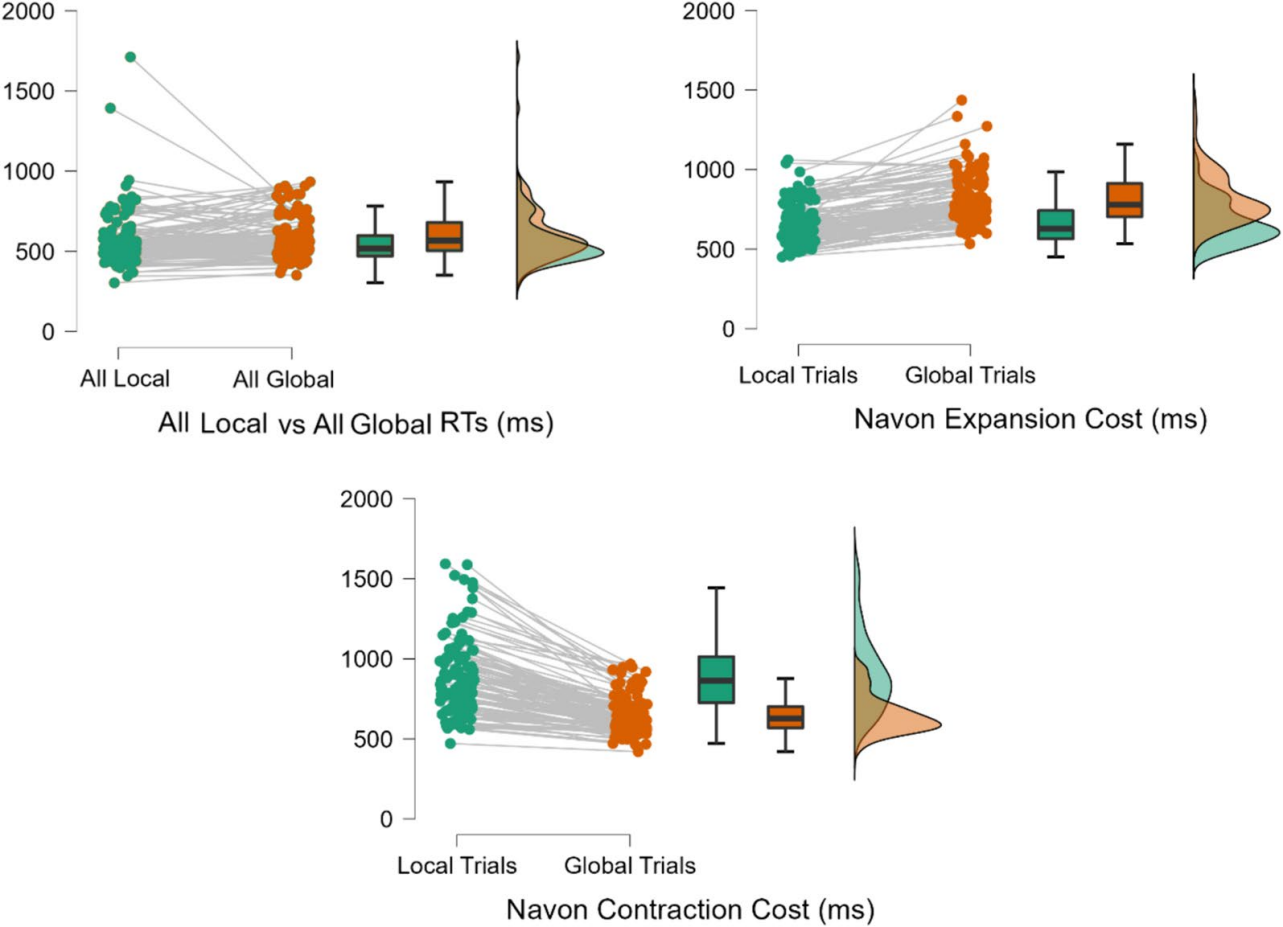
Comparative performance slopes for Navon tasks data by participant. Performance slopes across the stated trial-type comparisons are shown for each participant, as well as boxplots and distributions for sample performance under each working memory load. Performance for target-local trials is shown in green, while performance in target-global trials is shown in orange

For analysis of Navon RT performance, all comparisons were made using both frequentist and Bayesian repeated-measures t-tests using the default priors in JASP Version 0.18; Bayes factors were interpreted per the guidelines in Andraszewicz et al. (2014). All comparisons are depicted visually in Fig. 4. Comparison of mean RTs in the Navon All-Global and All-Local blocks indicated anecdotal evidence in favour of the null hypothesis of no difference between these conditions at the group level; *t*(106) = 1.858, *p* = 0.066, BF_10_ = 0.561, *d* = 0.18 (95% CI: [− 0.01, 0.37]). However, given the individual-differences nature of this research design, and the previous finding that older adults tend to prefer a narrower breadth of attention (Lawrence et al., 2018), this non-replication of classical global precedence effects for Navon figures is not of concern (see also Yovel et al., 2001). Scores in the Navon All-Global and All-Local conditions were correlated (*r*_*s*_ = 0.640, *p* < 0.001, 95% CI: [0.511, 0.741]). This confirmed the appropriateness of calculating the Navon Preference Score to partial out other sources of between-participant variance such as motor RT and isolate each participant’s relative ability to instantiate these two different breadths of attention.

Conversely, for the Navon Expansion task, there was extreme evidence in favour of the alternative hypothesis of a significant difference between majority target-local and minority target-global conditions; *t*(106) = -14.294, *p* < 0.001, BF_10_ = 2.08e + 23, *d* = − 1.38 (95% CI: [− 1.64, − 1.11]). Similarly, for the Navon Contraction task, there was extreme evidence in favour of the alternative hypothesis of a significant difference between majority target-global and minority target-local conditions; *t*(106) = 15.866, *p* < 0.001, BF_10_ = 3.54e + 26, *d* = 1.53 (95% CI: [1.25, 1.81]). Both effects were in the expected direction, such that performance was faster in the majority trial condition of each block than the minority one. This indicated that these results are consistent with the processes which these tasks were intended to operationalize. For the Navon Expansion score, slowed responses to target-global figures indicate a time cost associated with expanding the spatial scope of attention from the local to the global level. Conversely, for the Navon contraction score, longer response times for target-local figures are consistent with the time taken to contract attention from the global to the local level. Navon expansion and contraction scores were not significantly correlated (*r*_*s*_ = − 0.159, *p* = 0.108, 95% CI: [− 0.341, 0.035]), supporting the idea that they gauged two separate constructs rather than indexing a more general factor of attentional resizing ability.

### Reliability analyses

Individual-differences designs require a high level of rank-order reliability (the ability to stably rank participants based on performance). This is an important consideration because reliability places an upper constraint on the strength of association that can be observed between two measures (Hedge et al., 2018; Spearman, 1910). Reliability analyses were therefore conducted for the four measures derived from the Navon tasks in this study. Analyses were performed using the R package *splithalf* (Parsons, 2020), which uses a permutation-based calculation of the correlation between scores derived from two halves of total trials (e.g., odd versus even trials) for all participants in the sample. This tool calculates the correlation between the two halves of trials over 5000 random splits of the trials. This approach provides a mean estimate of split-half reliability, as well as a 95% confidence interval around that estimate. Navon Preference, Contraction, and Expansion reliability estimates were calculated for the RT-based difference score estimates. Reliability for all three measures was excellent (see Table 4), significantly above the recommended minimum of 0.7 recommended by Hedge et al. (2018), indicating that they were appropriate for use as predictors in the planned regressions.

**Table 4:** Spearman-Brown Corrected Reliability Estimates for Navon and Visual Search Tasks

Task	*r*_SB_^a^	95% CI	
		Lower	Upper
Navon preference	0.89	0.83	0.93
Navon contraction	0.92	0.89	0.95
Navon expansion	0.85	0.79	0.9

Navon All Global and All Local reliability estimates are calculated for mean RTs. Navon Contraction and Expansion reliability estimates are calculated for RT-based difference score estimates

^a^Spearman-Brown corrected reliability estimate

### Regression analyses

#### Screening and transformations

Prior to conducting regressions, the planned analyses were screened for multivariate outliers using Mahalanobis distance, resulting in removal of three further participants from the regression analyses. Although the final sample size used in these regressions was *n* = 104, lower than the intended *n* of 129, post hoc power analysis using G*Power (Faul et al., 2007) indicated that observed power with this sample size was still approximately 92%.

Shapiro–Wilk tests indicated that the distribution of UFOV Subtasks 2 (*W* = 0.645, *p* < 0.001) and 3 (*W* = 0.958, *p* = 0.002) scores violated the assumption of normality. Before transformation, other assumptions of regression (i.e., linearity, homoscedasticity, independence of residuals, absence of multivariate outliers) were checked for both regressions. These were not problematic for Subtask 3, so regression was performed upon this criterion variable without transformation in the main text. However, Subtask 2 conformed better to the assumptions of regression following a square-root transformation, so analysis using this transformed variable is reported in the main text. Importantly, however, all results remained identical when performing the regression on the untransformed Subtask 2 results (see Supplementary Materials).

#### Bayesian linear regressions

When planning the regression analyses, we preferred Bayesian regression over a frequentist approach because the purpose of this study is exploratory: instead of determining the portion of variance in UFOV performance accounted for by deployments of attentional breadth, we instead sought to assess the strength of the evidence for or against these deployments being implicated in task performance in the first place. The advantage of Bayesian regression in this scenario is that it permits model comparison to evaluate which predictor or combination of predictors receives the best support. Additionally, we accounted in our regression models for two covariates which are likely to affect UFOV performance: age and screen size. Age is known to influence UFOV performance, such that older participants perform more poorly on the task than younger ones (Bédard et al., 2016). Further, although stimulus sizing was not correlated with task performance on individual metrics of interest, we still elected to include screen size as a covariate in our regression models in case it interacted with our attentional breadth metrics as a predictor of UFOV performance.

Here, therefore, two Bayesian linear regression analyses were therefore performed in JASP 0.18 which compared a null model containing age and display size with an alternative model using three predictors: Navon Preference Score, Navon Expansion Score, and Navon Contraction Score. These regressions used all preset conditions in JASP 0.18, except that uniform model priors were specified, and Bayes factors are interpreted according to the guidelines in Andraszewicz et al. (2014). The outcome variable of the first regression model was Subtask 2 threshold, while for the second model it was Subtask 3 threshold. The goal of these regressions was to assess whether any aspects of attentional breadth predicted UFOV subtask performances over and above the expected impacts of age and stimulus sizing.

For the regression upon transformed Subtask 2 scores, the best-performing model of the observed data included only Navon Contraction score (Table 5). These data were 13.8 times more likely under the model containing Navon Contraction score as a predictor, compared to the null model, meaning that the alternative model received strong support. Additionally, examination of posterior summaries of coefficients (Table 6) indicated that Navon Contraction score was the only predictor which had strong support for an increased posterior probability (0.936, BF_inclusion_ = 14.743) of inclusion in the regression model. Conversely, posterior summaries of coefficients did not support the retention of either Navon Preference (BF_inclusion_ = 0.423) or Navon Expansion (*BF*_inclusion_ = 0.530) as predictors of Subtask 2 scores.

**Table 5:** Bayesian Linear Regression Model Comparison for Transformed UFOV Subtask 2 Scores

Model Terms	*P*(M)	*P*(M|data)	*BF*_M_	*BF*_10_	*R*^2^
Null Model	0.125	0.031	0.224	1.000	0.006
Navon Contraction	0.125	0.429	5.250	13.800	0.087
Navon Contraction + Navon Expansion	0.125	0.231	2.098	7.424	0.095
Navon Preference + Navon Contraction	0.125	0.180	1.536	5.794	0.090
Navon Preference + Navon Contraction + Navon Expansion	0.125	0.097	0.755	3.136	0.097
Navon Preference	0.125	0.014	0.099	0.449	0.010
Navon Expansion	0.125	0.012	0.089	0.402	0.008
Navon Preference + Navon Expansion	0.125	0.006	0.042	0.194	0.011

All models including the null model include the effects of age and screen size. *BF*_10_ calculated relative to null model

**Table 6:** Posterior Summaries of Coefficients for Alternative Bayesian Linear Regression Model for Transformed UFOV Subtask 2 Scores

Coefficient	*P*(incl)	*P*(incl|data)	*BF*_inclusion_	*M*	*SD*	95% CI	
						Lower	Upper
Intercept	–	–	–	6.621	0.211	6.219	7.049
Screen Size	–	–	–	− 0.001	0.004	− 0.008	0.007
Age	–	–	–	0.028	0.044	− 0.052	0.123
Navon Preference	0.500	0.297	0.423	0.000	0.001	− 0.004	0.001
Navon Contraction	0.500	0.936	14.743	0.003	0.001	0.000	0.006
Navon Expansion	0.500	0.346	0.530	0.001	0.001	− 0.001	0.005

Prior and posterior inclusion probabilities are not reported for screen size and age, as these terms are specified as part of the null model

For the regression upon Subtask 3 scores, the best-performing model of the observed data included only Navon Expansion score (Table 7). These data were 2.732 times more likely under the model containing Navon Contraction score as a predictor, compared to the null model, meaning that the alternative model received only anecdotal support. However, examination of posterior summaries of coefficients (Table 8) indicated that Navon Expansion score did receive moderate support for an increased posterior probability (0.751, BF_inclusion_ = 3.017) of inclusion in the regression model. Conversely, posterior summaries of coefficients did not support the retention of either Navon Preference (BF_inclusion_ = 0.302) or Navon Contraction (BF_inclusion_ = 0.472) as predictors of Subtask 3 scores.

**Table 7:** Bayesian Linear Regression Model Comparison for UFOV Subtask 3 Scores

Model Terms	*P*(M)	*P*(M|data)	*BF*_M_	*BF*_10_	*R*^2^
Null Model	0.125	0.141	1.145	1.000	0.210
Navon Expansion	0.125	0.384	4.367	2.732	0.250
Navon Contraction + Navon Expansion	0.125	0.197	1.714	1.399	0.261
Navon Preference + Navon Expansion	0.125	0.109	0.860	0.778	0.251
Navon Preference + Navon Contraction + Navon Expansion	0.125	0.061	0.453	0.432	0.261
Navon Contraction	0.125	0.047	0.342	0.331	0.215
Navon Preference	0.125	0.045	0.330	0.320	0.214
Navon Preference + Navon Contraction	0.125	0.017	0.120	0.119	0.219

All models including the null model include the effects of age and screen size. *BF*_10_ calculated relative to null model

**Table 8:** Posterior summaries of coefficients for alternative Bayesian linear regression model for UFOV subtask 3 scores

Coefficient	*P*(incl)	*P*(incl|data)	*BF*_inclusion_	*M*	*SD*	95% CI	
						Lower	Upper
Intercept	–	–	–	122.581	3.702	115.588	130.330
Screen Size	–	–	–	− 0.154	0.068	− 0.286	− 0.015
Age	–	–	–	3.667	0.809	2.176	5.415
Navon Preference	0.500	0.232	0.302	− 0.003	0.016	− 0.051	0.033
Navon Contraction	0.500	0.321	0.472	0.008	0.017	− 0.012	0.052
Navon Expansion	0.500	0.751	3.017	0.062	0.048	0.000	0.152

Prior and posterior inclusion probabilities are not reported for screen size and age, as these terms are specified as part of the null model

### Summary

Overall, the results of the regression analyses indicate that even after controlling for the covariates of age and screen size, UFOV performance was selectively associated with a different predictor for each subtask. Bayesian linear regression offered strong support for an association between Navon Contraction score and Subtask 2 performance, and anecdotal to moderate support for an association between Navon Expansion score and Subtask 3 performance.

## Discussion

Although UFOV has historically been understood as a measurement of attentional breadth, previous research seeking to establish whether this process is implicated in task performance has been inconclusive. This study aimed to systematically investigate possible relationships between two different aspects of attentional breadth (setting a specific breadth of attention, and resizing the attended region) and UFOV performance. Our findings indicate that even after controlling for the covariates of age and stimulus sizing, performances on UFOV Subtasks 2 and 3 are uniquely and selectively associated with different aspects of attentional breadth. UFOV Subtask 2 performance was associated with the efficiency of *contracting* attentional breadth, while Subtask 3 was associated with the efficiency of *expanding* attentional breadth.

### Selective implication of attentional breadth modulations

A key result from our study is that for both Subtasks 2 and 3, only one type of attentional breadth deployment was associated with task performance; furthermore, the associated type of deployment differed between the subtasks. This selectivity in the relationships suggests that these different attentional-breadth processes may actually be involved in how people complete the two tasks, rather than the relationships being mediated by an underlying cognitive process that is linked to performance on the tasks. In other words, if we had observed that all three measures of attentional breadth predicted performance on all of the subtasks, the most likely reason for that finding would be that both deployments of attentional breadth and UFOV performance draw on common underlying mechanisms such as executive function or attentional control. However, the associations we observed between Subtasks 2 and 3 were both task-selective and theoretically plausible, indicating that these attentional processes may be specifically implicated in different UFOV subtasks (see also Hoffman et al., 2005, for a discussion of how the cognitive and perceptual processes implicated in UFOV performance appear to differ across subtasks). In particular, the results suggest that for Subtask 2, participants may initially apply attention over a broad area to locate the peripheral stimulus and then contract it to the central stimulus to improve visual acuity and permit target identification. For Subtask 3, participants may tend to begin each trial with a small breadth of attention to identify the central stimulus and minimise the impact of crowding effects from the concentric rings of distractors, before expanding the breadth of attention in an attempt to identify the location of the peripheral target among distractors.

### Administering UFOV online

What do the findings from this study tell us about the possibility of conducting UFOV research online? UFOV is a task which is typically administered under highly controlled conditions, with factors such as stimulus sizing, viewing distance, display luminance, and environmental parameters kept constant across laboratory-based testing sessions. As explained in the Methods section, this study was conducted using an online remote-delivery format. We therefore chose to scale stimulus sizing to screen size for two reasons. Firstly, we did not want to fix stimulus sizing based on a ‘lowest common denominator’ screen size that would result in uniformly small displays; for instance, many modern ultraportable laptops only have an 11-inch screen size. Secondly, even if stimulus sizing was fixed across participants, differences in viewing angle and distance would have meant that stimuli did not actually subtend identical visual angles across participants, so this choice would not have achieved the intended standardisation. Instead, we imposed the eligibility criterion that participants needed to use a computer rather than a tablet or phone and included screen size as a covariate in regression analyses.

This approach was ultimately successful: roles for the resizing of attentional breadth in UFOV Subtasks 2 and 3 performance were found, even after controlling for screen sizing. However, we acknowledge that relinquishing control over task conditions could introduce random error variance which attenuated the observed relationships between UFOV and other cognitive variables. This issue may have accounted for the observed predictive strength of attentional expansion for Subtask 3 being relatively modest. Although screen size did not directly correlate with either UFOV Subtask 2 or 3 performance, the null model including age and screen size showed a substantial model *R*^*2*^ for Subtask 3. While these relationships were controlled for in the regression model and attentional expansion was still a significant predictor of performance, it is possible that other factors which are related to, but not fully represented by, screen size, attenuated the observed strength of this relationship. For instance, the beta weight for screen size in this regression was negative, such that participants could complete Subtask 3 at a lower display time threshold when the stimulus array was larger. Consequently, it is possible that the greater stimulus spacing resulted in a lowered impact of crowding effects in Subtask 3 for those with larger screens (Matas et al., 2014), making crowding susceptibility another source of individual variance in task performance.

Despite this, this study was sufficiently sensitive to detect relationships between UFOV and the ability to resize attentional breadth. It remains possible that the relationships observed here would be larger in magnitude when tested with the tighter experimental control over factors such as visual angle and luminance that is possible in a laboratory setting. Future research could test this possibility by comparing results obtained in laboratory-based versus online administrations of UFOV. Alternatively, if researchers are concerned about variance in screen size as a methodological issue, strategies are available to control for stimulus sizing: for instance, many web-based testing platforms allow for consistent sizing of stimuli using a tool such as a credit card as a reference point. However, the fact that associations were able to be detected suggests that UFOV can be adapted into online formats, which opens up new and more flexible practical options for future research using UFOV.

### Educational level of sample

We also note that UFOV performance in our sample was significantly better than that reported in a normative sample (Edwards et al., 2005a), such that participants could complete all three subtasks at lower display time thresholds and UFOV composite scores were markedly superior. This outcome does not appear to be attributable to variance in screen size inducing better performance, given the lack of correlation between UFOV scores and screen size. Instead, it may be linked to the relatively high level of education in our sample, as higher levels of education are known to be associated with superior UFOV performance (Edwards et al., 2005a). Equally, performance in our sample could reflect superior confidence and competency with the use of computer technology, a factor not controlled for in the dataset of Edwards et al., (2005a). UFOV performance is known to be positively associated with proficiency and positive attitudes towards computer use (Fazeli et al., 2013), and the self-selecting bias of recruiting online is likely to have ensured that our sample consisted of proficient computer users with positive attitudes towards computing. Three important aspects of our data lead us to believe that our sample performance neither reflected an anomalous administration of the UFOV task, nor presented a conceptual impediment to robustly detecting individual-differences relationships between UFOV and other constructs: (1) the internal *pattern* of correlations between UFOV subtasks remained similar in our sample compared to normative data (Edwards et al., 2005a), (2) there was a broad range of UFOV scores in the sample for both Subtasks 2 and 3, indicating that the task was still sensitive to individual differences in UFOV performance capability, and (3) our administration of the CFQ did not reveal a decreased incidence of cognitive failures (and by extension, lowered crash risk) compared to normative data for that task. Overall, we instead favour the interpretation that our sample’s performance is a valid reflection of a ‘best-case’ scenario for UFOV performance, studying highly educated adults who are proficient computer users.

### Interactions between predictors of UFOV performance

More broadly, we acknowledge that the amount of variance in UFOV performance explained by these regression models is relatively small. Ultimately, the purpose of this study is not to contend that attentional breadth is *responsible* for UFOV performance, but rather to demonstrate following a re-evaluation of previous work that modulations of attentional breadth play a *role* in task completion. A wide range of other cognitive and perceptual factors are known to be implicated in UFOV performance (for reviews, see Anstey et al., 2012; Aust et al., 2016; Woutersen et al., 2017), and other deployments of spatial attention such as visual search have also been linked to UFOV (Cosman et al., 2012b). Additionally, in accordance with contemporary understanding of UFOV as a processing speed measure, beta weights for the significant regression coefficients for both Subtasks 2 and 3 were positive—in other words, less efficient resizing of attention predicted poorer UFOV performance. Both working memory capacity (Anstey et al., 2012; Ball et al., 2007; Crisler et al., 2013) and processing speed (Wood & Owsley, 2014) are already well-known to be implicated in UFOV performance, and working memory capacity is also known to interact with the efficiency of attentional resizing (Goodhew, 2021). Subsequent research should therefore focus on establishing how deployments of spatial attention are moderated by other cognitive factors which bear upon the *efficiency* of attentional allocation during completion of UFOV.

## Conclusions

In conclusion, the efficiency of contracting attentional breadth from broad to narrow was selectively associated with individual differences in UFOV Subtask 2 performance, whereas the efficiency of expanding attentional breadth from narrow to broad was selectively associated with individual differences in UFOV Subtask 3 performance. This elucidates the long-assumed role of attentional breadth in UFOV, giving the task a clear grounding in specific deployments of spatial attention, and adding to the body of evidence which has attempted to explain how individuals complete the task. These findings also open the door to future research which can test whether training to improve the efficiency of resizing attentional breadth improves driver safety outcomes for older adults. This training could be particularly applicable in specific settings where modulations of the attended region might be expected, such as entering into traffic (Pietras et al., 2006), turning across intersections (Rusch et al., 2016), and driving in the presence of distractors (Wood et al., 2012). Similarly, this knowledge may inspire technological interventions which are designed to mitigate problems with the resizing of attentional breadth, for example early warning systems based on eye tracking (Carr & Grover, 2020) which detect moving objects which fall outside of an individual’s attended region.

### Supplementary Information

Additional file 1.

## Data Availability

A deidentified version of the dataset supporting the conclusions of this article is available in the OSF repository: https://osf.io/6ucp3/. This study was not preregistered.

## Abbreviations

CFQ: Cognitive Failures Questionnaire
UFOV: Useful Field of View task

## Footnotes

1 All visual angle dimensions given in the Methods section reflect stimulus sizing when presented on a 23-inch 1980 × 1020 monitor at a viewing distance of 60 cm. As described above, stimulus sizing could not be controlled for, such that the degrees of visual angle subtended by each object remained constant across participants. Instead, stimulus sizes were scaled to occupy the same percentage of screen dimensions across devices.
