# Supplementary material for: Different deployments of attentional breadth selectively predict UFOV task performance in older adults
Source: Cogn Res Princ Implic. 2024 Jun 26;9:42. doi: 10.1186/s41235-024-00569-3 (PMC11208374; doi:10.1186/s41235-024-00569-3)
Supplement: Supplementary file 1 — Additional file 1. [file 41235_2024_569_MOESM1_ESM.docx]

**Supplementary Materials**

**Running Orders**

Eight running orders were used in this experiment to minimise the possibility of order effects. The experiment had three overarching components: UFOV, Navon at set attentional breadth (i.e., All Global and All Local blocks), Navon resizing (i.e., Expansion and Contraction blocks). The running orders consisted of different combinations of these three components: for the two Navon components, the order of the blocks could change across running orders, but the blocks were never intermixed across the components (e.g. there was no running order that went Navon All Global 🡪 Navon Expansion 🡪 Navon All Local 🡪 Navon Contraction). The eight running orders were as follows:

1. UFOV 🡪 Navon All Global 🡪 Navon All Local 🡪 Navon Contraction 🡪 Navon Expansion
2. UFOV 🡪 Navon All Local 🡪 Navon All Global 🡪 Navon Contraction 🡪 Navon Expansion
3. UFOV 🡪 Navon All Global 🡪 Navon All Local 🡪 Navon Expansion 🡪 Navon Contraction
4. UFOV 🡪 Navon All Local 🡪 Navon All Global 🡪 Navon Expansion 🡪 Navon Contraction
5. Navon All Global 🡪 Navon All Local 🡪 Navon Contraction 🡪 Navon Expansion 🡪 UFOV
6. Navon All Local 🡪 Navon All Global 🡪 Navon Contraction 🡪 Navon Expansion 🡪 UFOV
7. Navon All Global 🡪 Navon All Local 🡪 Navon Expansion 🡪 Navon Contraction 🡪 UFOV
8. Navon All Local 🡪 Navon All Global 🡪 Navon Expansion 🡪 Navon Contraction 🡪 UFOV

**Navon RT Reanalysis: Existing Datasets**

To inform the number of trials used for our four Navon task designs, we reanalysed data reported in the supplementary materials of Goodhew and Plummer (2019) for the All Global and All Local tasks, as well as an unpublished dataset for an attentional breadth contraction task. The goal of this process was to identify a number of trials which would maximise the *reliability* of our experimental design. Individual-differences designs require a high level of rank-order reliability (the ability to stably rank participants based on performance). This is an important consideration because reliability places an upper constraint on the strength of association that can be observed between two measures (Hedge et al., 2018; Spearman, 1910).

Reliability for the measures calculated for these tasks were estimated with the R package splithalf (Parsons, 2020), which uses a permutation-based calculation of the correlation between scores derived from two halves of total trials (e.g., odd versus even trials) for all participants in the sample. This tool calculates the correlation between the two halves of trials over 5000 random splits of the trials. This approach provides a mean estimate of split-half reliability, as well as a 95% confidence interval around that estimate.

Results indicated that reliability converged on acceptable values after only 20 trials for the All Global and All Local tasks (Figure 2). For the difference score measure derived from the attentional contraction task, reliability demonstrated the best combination of a high Spearman-Brown estimate and narrow confidence interval at around 160 trials. Therefore, these trial numbers were used in our experiment design.

**Figure A1**

*Reliability Estimates for Navon Contraction Difference Score Data by Number of Trials (Goodhew & Plummer, 2019)*

**
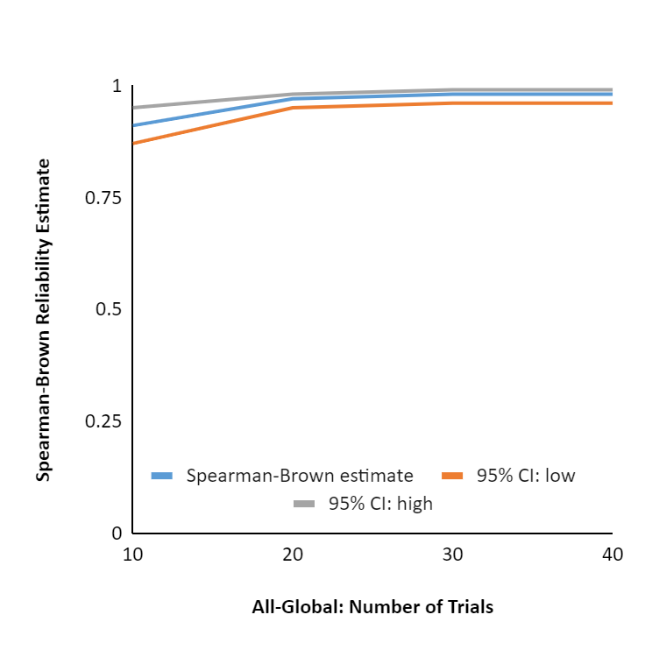

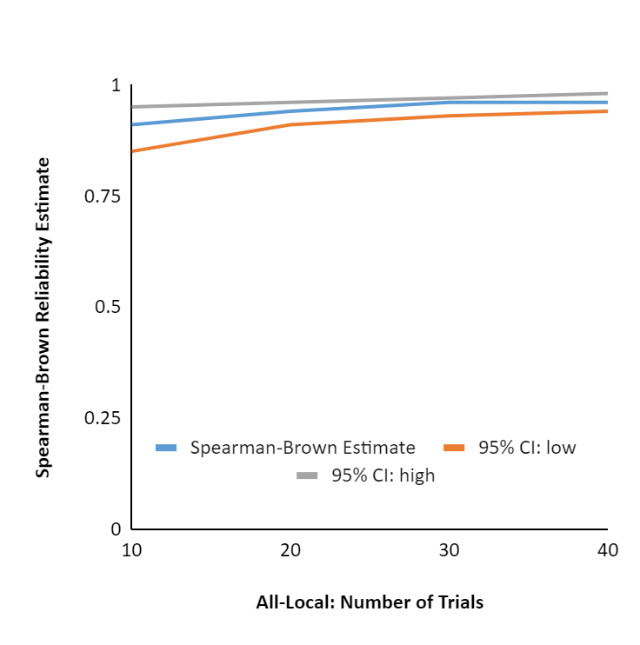
**

**Figure A2**

*Reliability Estimates for Navon Contraction Difference Score Data by Number of Trials (Unpublished Dataset)*


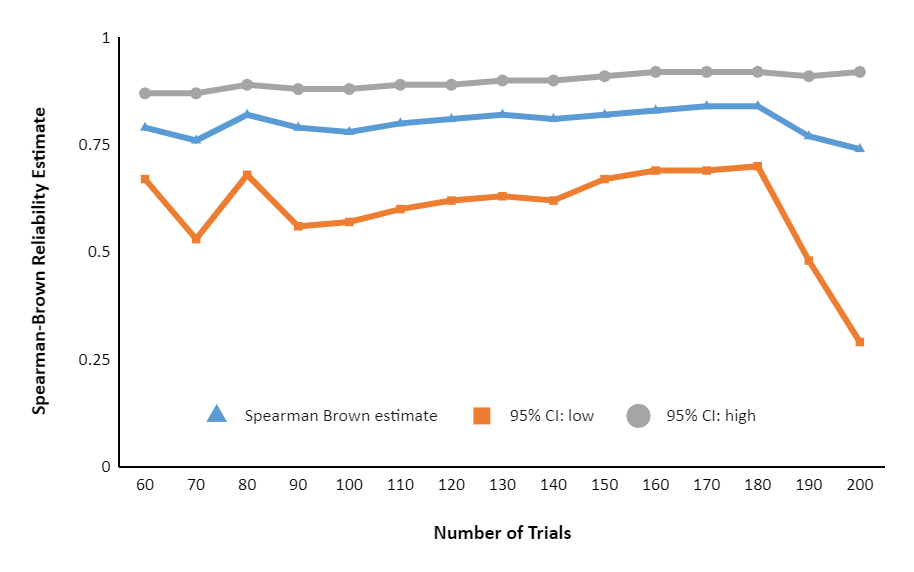


**Navon Accuracy Performance**

Accuracy performance was compared across task conditions to assess whether any speed-accuracy trade-offs occurred. All comparisons were performed using both frequentist and Bayesian repeated-measures t-tests using the default priors in JASP Version 0.17 (2023); Bayes factors were interpreted per the guidelines in Andraszewicz et al. (2014). Comparison of mean accuracy in the Navon All-Global and All-Local blocks indicated moderate evidence in favour of the null hypothesis of no difference between these conditions; *t*(106) = 0.606, *p* = .546, BF_10_ = 0.128, *d* = 0.06 (95% CI: [-0.13, 0.25]). Conversely, for the Navon Expansion task, there was extreme evidence in favour of the alternative hypothesis of a significant difference in accuracy between majority target-local and minority target-global conditions; *t*(106) = 4.165, *p* < .001, BF_10_ = 273.4, *d* = 0.40 (95% CI: [0.20, 0.59]). Similarly, for the Navon Contraction task, there was extreme evidence in favour of the alternative hypothesis of a significant accuracy difference between majority target-global and minority target-local conditions; *t*(106) = -11.575, *p* < .001, BF_10_ = 3.05e+17, *d* = -1.12 (95% CI: [-1.35, -0.88]). These effects were not indicative of a speed-accuracy trade-off – for both resizing tasks, accuracy in the minority condition was lower, corresponding to slower RTs in the minority conditions. This indicates that participants genuinely found resizing their attention for minority trials more challenging, rather than using lower accuracy as a strategy to perform faster on majority trials.

**Correlations between Screen Size and Performance on Key Metrics**

Correlations between screen size and UFOV subtask performance are reported in the main body. Correlations between key Navon task measures and screen size uniformly did not attain significance; these are reported in Table A1.

**Table A1**

*Correlations between Screen Size and Navon Task Performance*

|  | Navon Preference | Navon Expansion | Navon Contraction |
| --- | --- | --- | --- |
| Screen Size | .058  [-.133, .245] | .049  [-.142, .236] | -.028  [-.217, .163] |

*Note*. Spearman correlations reported.

**Subtask 2 Regression Analysis – Untransformed Data**

For the Bayesian linear regression upon transformed Subtask 2 scores, the best-performing model of the observed data included only Navon Contraction score (Table A2). These data were 16.0 times more likely under the model containing Navon Contraction score as a predictor, compared to the null model, meaning that the alternative model received strong support. Additionally, examination of posterior summaries of coefficients (Table A3) indicated that Navon Contraction score was the only predictor which had strong support for an increased posterior probability (0.944, *BF*_inclusion_ = 16.827) of inclusion in the regression model. Conversely, the addition of Navon Preference and Navon Expansion scores to Navon Contraction score as a predictor did not result in substantial improvements to model *R*^2^, and posterior summaries of coefficients did not support the retention of either Navon Preference (*BF*_inclusion_ = 0.297) or Navon Expansion (*BF*_inclusion_ = 0.335) as predictors of Subtask 2 scores.

**Table A2**

*Bayesian Linear Regression Model Comparison for Untransformed UFOV Subtask 2 Scores*

| Model Terms | *P*(M) | | *P*(M\|data) | | | *BF*_M_ | | *BF*_10_ | | *R*² | |  |
| --- | --- | --- | --- | --- | --- | --- | --- | --- | --- | --- | --- | --- |
| Null Model |  | 0.125 |  | 0.028 |  | | 0.198 |  | 1.000 |  | 0.000 |  |
| Navon Contraction |  | 0.125 |  | 0.441 |  | | 5.520 |  | 16.011 |  | 0.085 |  |
| Navon Contraction + Navon Expansion |  | 0.125 |  | 0.224 |  | | 2.017 |  | 8.123 |  | 0.092 |  |
| Navon Preference + Navon Contraction |  | 0.125 |  | 0.184 |  | | 1.582 |  | 6.696 |  | 0.088 |  |
| Navon Preference + Navon Contraction + Navon Expansion |  | 0.125 |  | 0.095 |  | | 0.735 |  | 3.450 |  | 0.094 |  |
| Navon Preference |  | 0.125 |  | 0.012 |  | | 0.088 |  | 0.450 |  | 0.005 |  |
| Navon Expansion |  | 0.125 |  | 0.011 |  | | 0.077 |  | 0.394 |  | 0.002 |  |
| Navon Preference + Navon Expansion |  | 0.125 |  | 0.005 |  | | 0.037 |  | 0.192 |  | 0.005 |  |

*Note*. All models including the null model include the effects of age and screen size. *BF*_10_ calculated relative to null model.

**Table A3**

*Posterior Summaries of Coefficients for Alternative Bayesian Linear Regression Model for Untransformed UFOV Subtask 2 Scores*

| Coefficient | | *P*(incl) | |  | *P*(incl\|data) | | | *BF*_inclusion_ | | *M* | *SD* | 95% CI | |
| --- | --- | --- | --- | --- | --- | --- | --- | --- | --- | --- | --- | --- | --- |
|  |  |  |  |  |  |  |  |  |  |  |  | Lower | Upper |
| Intercept |  | - |  |  |  | - |  | | - | 48.655 | 3.828 | 41.631 | 56.628 |
| Screen Size |  | - |  |  |  | - |  | | - | -0.001 | 0.067 | -0.132 | 0.132 |
| Age |  | - |  |  |  | - |  | | - | 0.137 | 0.801 | -1.434 | 1.712 |
| Navon Preference |  | 0.500 |  |  |  | 0.297 |  | | 0.423 | -0.004 | 0.018 | -0.052 | 0.037 |
| Navon Contraction |  | 0.500 |  |  |  | 0.944 |  | | 16.827 | 0.060 | 0.026 | 0.000 | 0.101 |
| Navon Expansion |  | 0.500 |  |  |  | 0.335 |  | | 0.503 | 0.009 | 0.025 | -0.032 | 0.072 |

*Note.* Prior and posterior inclusion probabilities are not reported for screen size and age, as these terms are specified as part of the null model.

**References**

Andraszewicz, S., Scheibehenne, B., Rieskamp, J., Grasman, R., Verhagen, J., & Wagenmakers, E.-J. (2014). An Introduction to Bayesian Hypothesis Testing for Management Research. *Journal of Management*, *41*(2), 521-543. https://doi.org/10.1177/0149206314560412

Faul, F., Erdfelder, E., Lang, A. G., & Buchner, A. (2007). G*Power 3: A flexible statistical power analysis program for the social, behavioral, and biomedical sciences. *Behavior Research Methods*, *39*(2), 175-191. https://doi.org/10.3758/Bf03193146

Goodhew, S. C., Dawel, A., & Edwards, M. (2020). Standardizing measurement in psychological studies: On why one second has different value in a sprint versus a marathon. *Behavior Research Methods*. https://doi.org/10.3758/s13428-020-01383-7

Goodhew, S. C., & Plummer, A. S. (2019). Flexibility in resizing attentional breadth: Asymmetrical versus symmetrical attentional contraction and expansion costs depends on context. *Quarterly Journal of Experimental Psychology*, *72*(10), 2527-2540. https://doi.org/10.1177/1747021819846831

Hedge, C., Powell, G., & Sumner, P. (2018). The reliability paradox: Why robust cognitive tasks do not produce reliable individual differences. *Behavior Research Methods*, *50*(3), 1166-1186. https://doi.org/10.3758/s13428-017-0935-1

Parsons, S. (2020). *splithalf: Calculate Task Split Half Reliability Estimates*. In (Version 0.7.1)

Spearman, C. (1910). Correlation Calculated from Faulty Data. *British Journal of Psychology*, *3*, 271-295. https://doi.org/DOI 10.1111/j.2044-8295.1910.tb00206.x

Tabachnick, B., & Fidell, L. L. S. (2013). *Using Multivariate Statistics* (Vol. 983).
